# Exposure–response analyses of cabozantinib in patients with metastatic renal cell cancer

**DOI:** 10.1186/s12885-022-09338-1

**Published:** 2022-03-02

**Authors:** Stefanie D. Krens, Nielka P. van Erp, Stefanie L. Groenland, Dirk Jan A. R. Moes, Sasja F. Mulder, Ingrid M. E. Desar, Tom van der Hulle, Neeltje Steeghs, Carla M. L. van Herpen

**Affiliations:** 1 Department of Pharmacy, Radboud Institute for Health Sciences, Radboud University Medical Center, Geert Grooteplein Zuid 10, 6525 GA Nijmegen, The Netherlands; 2 Division of Medical Oncology, Department of Clinical Pharmacology, The Netherlands Cancer Institute-Antoni Van Leeuwenhoek, Plesmanlaan 121, 1066 CX Amsterdam, The Netherlands; 3 Department of Clinical Pharmacy and Toxicology, Leiden University Medical Center, Albinusdreef 2, 2333 ZA Leiden, The Netherlands; 4 Department of Medical Oncology, Radboud Institute for Health Sciences, Radboud University Medical Center, Geert Grooteplein Zuid 10, 6525 GA Nijmegen, The Netherlands; 5 Department of Medical Oncology, Leiden University Medical Center, Albinusdreef 2, 2333 ZA Leiden, The Netherlands

**Keywords:** Cabozantinib, Renal cell carcinoma, Pharmacokinetics, Exposure, Response, Survival, Toxicity, Pharmacodynamics

## Abstract

**Aim:**

In the registration trial, cabozantinib exposure ≥ 750 ng/mL correlated to improved tumor size reduction, response rate and progression free survival (PFS) in patients with metastatic renal cell cancer (mRCC). Because patients in routine care often differ from patients in clinical trials, we explored the cabozantinib exposure–response relationship in patients with mRCC treated in routine care.

**Methods:**

Cabozantinib trough concentrations (C_min_) were collected and average exposure was calculated per individual. Exposure–response analyses were performed using the earlier identified target of C_min_ > 750 ng/mL and median C_min_. In addition, the effect of dose reductions on response was explored. PFS was used as measure of response.

**Results:**

In total, 59 patients were included:10% were classified as favourable, 61% as intermediate and 29% as poor IMDC risk group, respectively. Median number of prior treatment lines was 2 (0–5). Starting dose was 60 mg in 46%, 40 mg in 42% and 20 mg in 12% of patients. Dose reductions were needed in 58% of patients. Median C_min_ was 572 ng/mL (IQR: 496–701). Only 17% of patients had an average C_min_ ≥ 750 ng/mL. Median PFS was 52 weeks (95% CI: 40–64). No improved PFS was observed for patients with C_min_ ≥ 750 ng/mL or ≥ 572 ng/ml. A longer PFS was observed for patients with a dose reduction vs. those without (65 vs. 31 weeks, *p* = .001). After incorporating known covariates (IMDC risk group and prior treatment lines (< 2 vs. ≥ 2)) in the multivariable analysis, the need for dose reduction remained significantly associated with improved PFS (HR 0.32, 95% CI:0.14–0.70, *p* = .004).

**Conclusion:**

In these explorative analyses, no clear relationship between increased cabozantinib exposure and improved PFS was observed. Average cabozantinib exposure was below the previously proposed target in 83% of patients. Future studies should focus on validating the cabozantinib exposure required for long term efficacy.

**Supplementary Information:**

The online version contains supplementary material available at 10.1186/s12885-022-09338-1.

## Introduction

Renal cell carcinoma (RCC) is in the top ten of most common cancers in high income countries. In Europe, its incidence is estimated at approximately 140.000 cases a year [1, 2]. RCC consists of a heterogenous group of malignant neoplasms with distinct pathological features and different molecular alterations [3, 4]. In more than 50% of all RCC, a somatic mutation or epigenetic alteration of the von Hippel-Lindau tumour suppressor gene is present that leads to its inactivation and subsequent accumulation of the transcriptional regulatory molecule hypoxia-inducible factor alpha (HIFα) [5, 6]. Accumulation of HIFα can cause upregulation of hypoxia-response genes, including the vascular endothelial growth factor (VEGF), the platelet derived growth factor (PDGF) and the hepatocyte growth factor receptor (MET) [6]. The treatment armamentarium for mRCC has expanded greatly over the past two decades with the introduction of oral tyrosine kinase inhibitors (TKI) directed against VEGF receptors (VEGFR), inhibitors of the mammalian target of rapamycin pathway, immune checkpoint inhibitors (ICI) directed against the programmed death-1 receptor, PD-1 ligand or cytotoxic T-lymphocyte-associated protein 4 and the HIF2α inhibitor belzutifan [7]. More recently, specific combinations of ICI + ICI and ICI + TKI demonstrated increased response rates and progression free survival (PFS) and are currently recommended as first line treatment options [8, 9].

Cabozantinib is an oral multitarget TKI that is a potent inhibitor of VEGFR-2, MET and the Tyro3, Axl and Mer (TAM) family of receptor kinases [10]. In 2016 cabozantinib was initially approved for second line treatment in patients who received prior TKI therapy. Based on the results of the CABOSUN trial, cabozantinib was added as fist line treatment option in poor/intermediate IMDC risk mRCC in 2019 [11, 12]. Recently, both U.S. Food and Drug Administration (FDA) and European Medicines Agency (EMA) approved the combination of cabozantinib + nivolumab for patients with advanced RCC based on the CheckMate 9ER study [13]. Furthermore, cabozantinib is one of the few TKIs which has shown intracranial activity in patients with brain metastases [14]. Cabozantinib has also been approved for treatment of hepatocellular carcinoma, advanced differentiated thyroid cancer and metastatic medullary thyroid cancer. Currently, cabozantinib is being studied in multiple combination treatment regimens [15].

The recommended starting dose of single-agent cabozantinib is 60 mg once daily (OD). However, in the registration studies 40–62% of patients required a dose reduction [16, 17]. For other VEGFR inhibitors used for RCC (i.e. sunitinib, pazopanib, axitinib) exposure–response analyses have revealed a clear exposure target above which improved clinical efficacy can be expected [18–23]. For cabozantinib, the exposure–response relation has been investigated in patients with RCC in the phase III METEOR trial (*n*= 330). The average exposure reached with the standard starting dose of 60 mg OD was shown to be 1125 ng/mL and resulted in improved PFS, reduced tumour growth and increased overall response rate (ORR) compared to the exposure reached with 40 and 20 mg doses [24]. For the 40 mg dose with an average exposure of 750 ng/mL, only a modest 1.1 fold increased risk for disease progression was observed compared to average exposures of 1125 ng/mL. For the 20 mg dose with an average exposure of 375 ng/ml, the risk had increased to 1.4-fold. Based on only a modestly improved efficacy at a dose of 60 mg but poor tolerability, we proposed an exposure of > 750 ng/mL as target exposure for optimal treatment outcome. Similar to other tyrosine kinase inhibitors, cabozantinib shows a large between-patient variability of ~ 40% and a within-patient variability of ~ 30% [24–26]. This large between-patient variability together with the proposed target exposure for beneficial efficacy, makes cabozantinib a suitable candidate for dose optimisation based on measured drug levels, also known as therapeutic drug monitoring (TDM). In a previous analysis from our group, we investigated the exposure toxicity relationship of cabozantinib in a limited number of patients with salivary gland cancer and patients with RCC [27]. The best tolerated exposure was ~ 600 ng/mL in both tumour types and considerably lower than the proposed target, which questions the feasibility of a target exposure ≥ 750 ng/mL. Moreover, this threshold has been established in patients included in the phase III trial. These patients are often a poor representation of the patients in routine clinical care, who are generally older, are heavily pre-treated, have a lower performance score and a higher number of comorbidities [28, 29].

The aim of this study was therefore to describe the exposure–response relationship for cabozantinib in a cohort of patients with RCC treated in routine care. The secondary aims were to assess the exposure-toxicity relationship, explore the exposure–response relationship for overall survival and describe cabozantinib pharmacokinetics. Finally, an algorithm to optimise cabozantinib treatment in clinical practice was designed.

## Material and methods

### Patient population and treatment patterns

For this retrospective observational study we collected clinical data and measured cabozantinib trough concentrations (C_min_) from patients treated with cabozantinib for mRCC in three Dutch hospitals (Netherlands Cancer Institute, Leiden University Medical Center and Radboud University Medical Center), between March 2017 and March 2021. Demographic, pathological, laboratory and prior systemic therapy data at start of cabozantinib treatment were retrospectively retrieved from the electronical health records. For cabozantinib treatment, information on starting dose, dose adjustments, dose interruptions, concomitant use of strong CYP3A4 inhibitors or inducers, and reason of discontinuation or adjustment were collected.

### Cabozantinib pharmacokinetics

Patients had plasma cabozantinib C_min_levels measured as part of routine care. For patients from the Radboudumc and the Leiden University Medical Center, a previously described validated high-performance liquid chromatography coupled with tandem mass spectrometry detection (UPLC-MS/MS) assay was used to determine total cabozantinib concentrations in plasma [30]. For patients treated in the Netherlands Cancer Institute a comparable UPLC-MS/MS method was used. Both methods were cross validated and showed comparable results.

Patients with at least one cabozantinib C_min_ level at steady-state were included. Steady-state was defined as cabozantinib treatment at the same dose level for 17 or more consecutive days, based on four times the half-life of cabozantinib which is approximately 4 days. Only samples measured at steady-state were included in the analysis. As this is a study on retrospectively collected data, no predefined sampling moments were set. However, therapeutic drug monitoring is well implemented in the participating clinics and the first measurement is usually performed approximately 4 weeks after treatment initiation or dose adjustment. For each sample, the date and time of last intake of cabozantinib and the date and time of the plasma sample collection were recorded. In case the sample was not collected 24 h after last intake, the trough concentration was estimated by log-linear extrapolation based on the elimination half-life and time after dose, as previously described by Wang et al. [31].

As cabozantinib has shown dose-proportional exposure over the range of 20 to 140 mg, evaluation of average cabozantinib exposure was performed by dose extrapolation. This procedure is described in detail in Supplementary method 1. For each patient the cabozantinib exposure at start dose level, at best tolerated dose and over the duration of treatment was calculated and compared to the 750 ng/mL threshold. Furthermore, between-patient and within-patient variability of cabozantinib were assessed at the 40 mg dose level.

### Exposure response analysis

Exposure response analyses were performed to assess if cabozantinib exposure was associated with treatment outcome. PFS was defined as the time between start of cabozantinib and discontinuation due to progressive disease or death. Patients who did not experience progressive disease or death were censored at the date of cabozantinib treatment discontinuation due to other causes or the date of last follow-up. Overall survival (OS) was defined as the time between start of cabozantinib and the date of death, or it was censored at the date of last follow-up. For cabozantinib exposure, the following exposure cut-off measures were used based on previous analysis [24]: C_min_ at start dose ≥ 750 ng/mL versus < 750 ng/mL, average C_min_ over the whole duration of treatment ≥ 750 ng/mL versus < 750 ng/mL. An analysis of C_min_ calculated over the first 90 days of treatment ≥ 750 ng/mL versus < 750 ng/mL was performed as sensitivity analysis, since this interval captures the influence of early dose adjustments and is considered long enough to attribute treatment benefit to cabozantinib exposure. Additionally, the relationship between C_min_ over the duration of treatment equal and above versus below the median C_min_ of the cohort and dose reduction relative to the starting dose yes/no in relation to PFS and OS was explored. Furthermore, the influence of the start dose (60 mg versus 40 mg) on PFS was examined. The influence of a start dose of 20 mg was not evaluated since patients starting at this lower dose are most likely in poor clinical condition. Finally, an explorative multivariable analysis for PFS was performed. Factors with a known or presumed correlation to outcome were included in multivariable analysis (i.e., IMDC risk group, prior lines of treatment (< 2 of ≥ 2) [32].

### Exposure toxicity analysis

Adverse events necessitating dose reduction were considered clinically relevant toxicities. Patients were divided in two groups: patients who received a dose reduction due to adverse events or patients without a dose reduction relative to their starting dose. The relationship between cabozantinib exposure and toxicity was assessed by comparing the cabozantinib C_min_ at the starting dose between both groups. The best tolerated dose level (BTD) was defined as the latest dose level before treatment discontinuation or at time of data cut off. In addition to toxicity, we compared the cabozantinib C_min_ at BTD between patients with and without a dose reduction to help define a therapeutic target window.

### Proposal for dose optimisation

Based on the results of the exposure–response analysis and the exposure-toxicity analysis, an algorithm to optimise cabozantinib treatment for patients in routine care was created.

### Statistical analysis

Baseline patient characteristics and cabozantinib treatment patterns were described using descriptive statistics. Differences in cabozantinib exposure between patient subgroups were tested with the Mann–Whitney U test. PFS and OS were estimated with the Kaplan–Meier method and differences between groups were examined by the log-rank test. Multivariable analysis was performed with Cox regression analysis. All statistical computations were performed in IBM SPSS statistics for Windows version 25.0 (IBM Corp, Armonk, NY, USA). No adjustments for multiplicity were made for subgroup analyses as these were considered exploratory.

## Results

### Patient population and treatment patterns

In total, 59 patients were included in this study. Baseline characteristics at start of cabozantinib treatment are presented in Table 1. Most patients had a tumour with clear cell histology (81.4%) and were classified to the intermediate IMDC risk group (61%). Cabozantinib was mainly given as second (33.9%) or third line (37.3%) treatment. The majority of patients were pre-treated with a VEGFR inhibitor (88.1%) and a considerable number had received both prior ICI and prior VEGFR inhibitor (64.4%).

**Table 1:** Baseline characteristics

Characteristic	Overall (*n* = 59)
**Age (years)**	63 (37–84)
**Male gender**	48 (81.4)
**Cell Histology**	
Clear cell	48 (81.4)
Papillary	6 (10.2)
Other	3 (5.1)
Missing	2 (3.4)
**IMDC**	
Favourable	6 (10.2)
Intermediate	36 (61.0)
Poor	17 (28.8)
**Weight (kg)**	80 (51–136)
**Albumin (g/l)**	36 (20–48)
**Nephrectomy (yes)**	39 (66.1)
**Number of lines of prior therapy for RCC**	2 (1–6)
0	2 (3.4)
1	20 (33.9)
2	22 (37.3)
≥ 3	15 (25.4)
**Prior VEGFR-I (yes)**	52 (88.1)
**Prior ICI (yes)**	43 (72.9)
**Prior ICI and prior VEGFR-I**	38 (64.4)

Data are presented as n (%) for categorical variables and median (range) for continuous variables

*Abbreviations*: *RCC* Renal cell carcinoma, *IMDC* International Metastatic RCC Database Consortium, *BMI* Body mass index, *VEGFR-I* Vascular endothelial growth factor receptor inhibitor, *ICI* Immune checkpoint inhibitor

Details of cabozantinib treatment are shown in Table 2. The cabozantinib starting dose was 60 mg for 46% patients, 40 mg for 42% patients and 20 mg for 12% patients. In 34 (58%) patients a dose reduction relative to the starting dose was needed due to toxicity. Six patients (10%) had a dose increase relative to their start dose. Median best tolerated dose was 40 mg. Alternative dose schedules at the BTD (other than 20, 40 or 60 mg OD) were used in 10 (17%) patients. Overall, median time on treatment was 34 weeks, with a median follow up of 44 weeks. Forty-one patients (70%) discontinued cabozantinib treatment: 29 (49%) due to progressive disease, nine (15%) due to toxicity and three (5%) for other reasons. None of the patients used strong CYP3A4 inhibitors or inducers during cabozantinib therapy.

**Table 2:** Cabozantinib treatment details

	**Overall (*n* = 59)**	**Dose reduction**^**b**^** (*n* = 34)**	**No dose reduction**^**b**^**(*n* = 25)**
**Treatment duration (weeks)**	34 (4–204)	46 (8–204)	23 (4–173)
**Average daily dose (mg)**	38 (12–60)	32 (12–56)	40 (20–60)
**Initiation dose (mg)**			
60	27 (46)	21 (62)	6 (24)
40	25 (42)	12 (37)	13 (52)
20	7 (12)	1 (3)	6 (24)
**Best tolerated dose in mg**	40 (10–60)	30 (10–50)	40 (20–60)
60	7 (12)	0 (0)	7 (28)
50^a^	2 (3)	1 (3)	1 (4)
40	27 (46)	12 (36)	15 (60)
26–31^a^	7 (12)	7 (21)	0
20	15 (25)	13 (38)	2 (8)
10^a^	1 (2)	1 (3)	0
**Treatment discontinuation**	41 (70)	19 (56)	22 (88)
Progressive disease	29 (49)	15 (43)	16 (54)
Toxicity	9 (15)	4 (11)	5 (20)
Other	3 (5)	2 (6)	1 (4)

Data are presented as n (%) for categorical variables and median (range) for continuous variables

^a^ cabozantinib dose reached via an alternative dosing schedule (e.g. 20 and 40 mg used alternately)

^b^ relative to the starting dose

### Cabozantinib pharmacokinetics

In total, 118 cabozantinib samples at steady-state were available with a median of 2 samples per patient (range 1–6). Between patient variability in C_min_ was 35.2% and the average within patient variability, assessed in patients with 2 or more samples (*n* = 37), was 22.8% (95%CI: 18.2–27.4). No clinically relevant decline in dose-normalized cabozantinib exposure was observed over time. The median exposure at starting dose was 745 ng/mL (Interquartile range (IQR) 559–942). Twenty-eight patients (47.5%) had an exposure above the proposed target threshold of ≥ 750 ng/mL at the start dose level. At the best tolerated dose level, median exposure was 543 ng/mL (IQR 467–739). The median average exposure over the duration of treatment was 572 ng/mL (IQR 496–701). Ten patients (16.9%) had an average exposure ≥ 750 ng/mL.

### Exposure response

#### Progression free survival

**Fig. 1:**
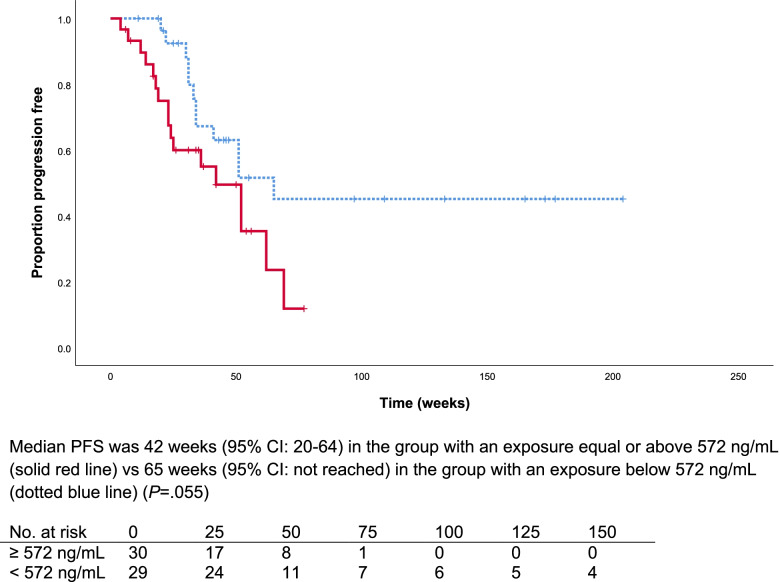
Kaplan–Meier curve of progression free survival for patients with an exposure above and below the median exposure over the duration of treatment (572 ng/mL)

**Fig. 2:**
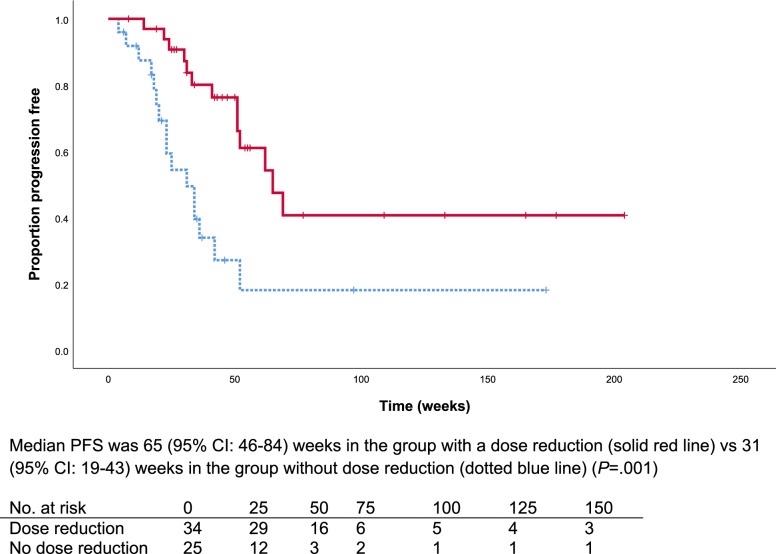
Kaplan–Meier curve of progression free survival for patients with and without a dose reduction

Median PFS overall was 52 weeks (95% CI: 40–64). No statistically significant difference in PFS was observed for patients with an average C_min_ ≥ 750 ng/mL compared to patients with an average C_min_ < 750 ng/mL (19 weeks (95% CI:0–40) vs. 52 weeks (95% CI: 34–70), respectively, *P* = 0.2). Also, no difference in PFS was observed in patients with a C_min_ at the starting dose ≥ 750 ng/mL compared to patients with a C_min_ < 750 ng/mL(52 weeks (95% CI: 32–72) vs 42 weeks (95% CI:17–66), respectively, *P* = 0.6)(Supplementary Fig. 1A and 1B). Similarly, no difference in PFS was observed between patients who started treatment with, 60 mg compared to those who started with 40 mg (Supplementary Fig. 1C) In addition, no difference was observed in the sensitivity analysis of patients with an average C_min_ ≥ 750 ng/mL over the first 90 days compared to patients with an average C_min_ < 750 ng/mL, *P* = 0.7 (Supplementary Fig. 1D). Moreover, a numerically non-significant longer PFS was observed for patients with an average C_min_ below the median of 572 ng/mL compared to patients with C_min_ ≥ 572 ng/mL; 65 weeks (95% CI: not reached) vs. 42 weeks (95% CI: 20–64) respectively, *P* = 0.055 (Fig. 1). Furthermore, a significantly increased PFS was observed for patients who received a dose reduction compared to patients who did not, 65 weeks (95% CI: 46–84) vs. to 31 weeks (95% CI: 19–43), respectively *P* = 0.001 (Fig. 2).

In multivariable analysis, having a dose reduction resulted in a 68% reduction in the risk of progression (hazard ratio (HR) 0.32 (95% CI: 0.14–0.70, *P* = 0.004), when IMDC risk group and previous lines of treatment (< 2 and ≥ 2) were taken into account (Supplementary Table 1).

#### Overall survival

Median follow up was 44 weeks (range 5–204) and during this follow up, 30 (51%) patients had died. Median OS was 64 weeks (95% CI: 51–77). No events occurred in patients with favourable IMDC risk score during follow up. Median OS was 67 weeks (95%CI: 59–75) in patients with intermediate risk and 41 weeks (95% CI: 34–48) in patients with poor risk. As the follow up for overall survival is relatively short in this study, explorative analyses for OS were performed only for the comparisons that resulted in survival differences for PFS (i.e. median exposure and dose reduction). Additionally, because of the large differences in survival time, the analyses were performed separately for intermediate and poor IMDC risk score. No difference was observed in OS for patients with an average cabozantinib exposure above the median value compared to patients with an exposure below the median value for both risk groups (66 weeks vs. not reached, *p* = 0.2 and 42 vs. 36 weeks, *P* = 0.6 respectively). For the intermediate risk patients, a longer survival was observed in patients who received a dose reduction (Not reached vs. 48 weeks, *P* = 0.002), whereas for poor risk patients the difference was minimal (42 vs. 36 weeks, *P* = 0.3 (Supplementary Figs. 2A-E).

### Exposure toxicity

**Fig. 3:**
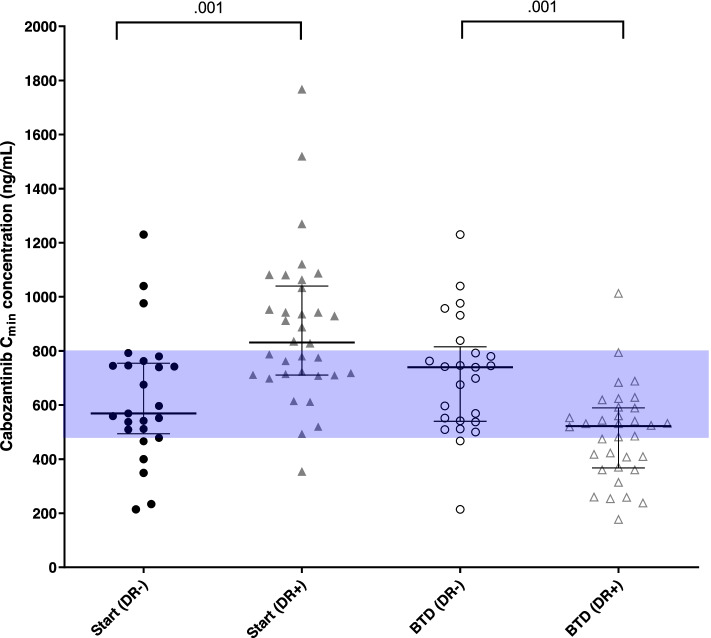
Scatterplots of the cabozantinib C_min_ levels at starting dose and at best tolerated dose for patients with and without a dose reduction. Abbreviations: DR, dose reduction. Whiskers show median and interquartile range (IQR). Shaded area represents the IQR of exposure reached at best tolerated dose of 40 mg

Patients who received a dose reduction had a significantly higher cabozantinib exposure at the start of therapy compared to patients that did not have a dose reduction, i.e. median C_min_ 831 ng/mL (IQR 711–1040) vs. 569 ng/mL (IQR 494–754), *P* = 0.001.Exposure at the BTD was lower in patients who received a dose reduction compared to patients who did not receive a dose reduction, i.e. median C_min_ 522 ng/mL (IQR 368–590) vs. 740 ng/mL (IQR 540–815), *P* = 0.001 (Fig. 3). Dose reductions were more prevalent in patients with an exposure ≥ 750 ng/mL at start dose compared to patients with an exposure < 750 ng/mL (78.6% vs. 38.7%, *P* = 0.003).

**Fig. 4:**
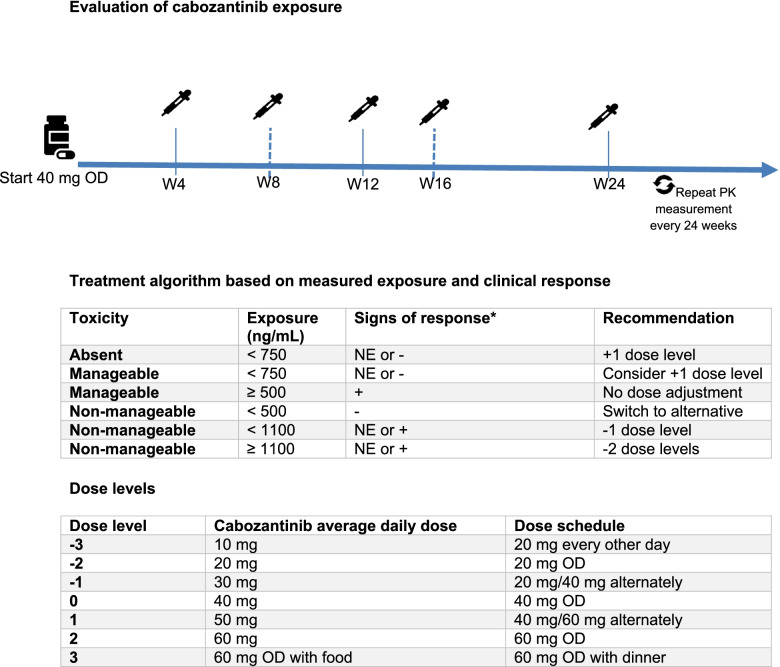
Proposed cabozantinib treatment algorithm. Patients start treatment at the dose of 40 mg once daily (OD). PK samples will be collected 4 and 12 weeks after start of treatment and every 24 weeks thereafter. The dose can be adjusted according to the treatment algorithm (4B) after checking for treatment adherence and drug-drug interactions. After dose adjustment, a new PK sample will be collected after 4 weeks. * Signs of response can be based on clinical symptoms, improvement of laboratory values (e.g. haemoglobin, albumin, calcium level) or based on radiological evaluation of tumour burden. Abbreviations: NE; not evaluable, OD; once daily

### Proposal for treatment optimisation

Figure 4 shows the proposed treatment optimisation strategy, combining clinical indicators of response and measured cabozantinib exposure. The algorithm was developed based on three main steps. The first step was the selection of the optimal starting dose. Based on the exposure-toxicity analyses, most patients have a tolerable exposure in the range between 500–800 ng/mL, which corresponds to a 40 mg dose. Therefore, 40 mg was selected as the most appropriate starting dose to avoid excessive toxicity. As the exposure–response analyses showed no clear incremental benefit with higher doses, the algorithm mainly focuses on attaining optimal tolerable exposure. Therefore, combined evaluation of signs of response, toxicity as well as cabozantinib exposure and suggestions for treatment adjustment were included as a second step. Alternative dosing schedules, e.g. 30 mg or 50 mg dose level, have been added to help patients achieve their individual optimal exposure in the narrow tolerability window. The third step includes evaluation 4 weeks after dose adjustment or the regular follow-up check every 24 weeks.

## Discussion

In this retrospective observational study, we investigated the exposure response relationship of cabozantinib in a group of unselected patients with mRCC treated with cabozantinib in routine care. Median PFS was approximately 52 weeks (12 months) and the median cabozantinib exposure over the duration of treatment was 572 ng/mL. No increased PFS or OS was observed for patients with a cabozantinib exposure above this median exposure compared to those with a lower exposure. Interestingly, patients who needed a dose reduction due to toxicity showed favourable PFS compared to patients without a dose reduction, even after correcting for IMDC risk score and number of prior treatment lines.

Previous real world studies have reported PFS ranging from 25 to 54 weeks, with an average of 35 weeks, which matches the PFS of 7.4 months (32 weeks) reported in the phase III METEOR study [17, 33–43]. The slightly longer PFS in our study could be the result of numerous factors, including tumour heterogeneity, prior treatment lines, especially prior ICI, and selection of patients eligible to be treated with cabozantinib.

Our observation of a longer PFS and OS for patients who required a dose reduction is in line with the findings of two previous real-world studies. Gan and colleagues reported decreased risk of time to treatment failure (TTF) and longer OS in patients who required a dose reduction compared to patients who did not in a cohort of patients with RCC treated with cabozantinib in the first to fourth line setting (HR: 0.37 and 0.46, respectively) [36]. Albiges and colleagues observed a remarkably longer OS in patients who required a dose reduction compared to patients without a dose reduction (17.5 vs 8.9 months) in the CABOREAL study [35]. This finding of an increased survival in patients requiring dose reductions has also been observed for the VEGFR inhibitors sunitinib, pazopanib and axitinib indicating that toxicity may serve as a surrogate marker for clinical response [23, 44–47]. The experienced toxicities in these patients may have a pharmacokinetic explanation (i.e. high drug exposure), but they could also be a reflection of a pharmacodynamic effect (i.e. potent inhibition of target pathways), or a combination of both. Previous studies with axitinib and sunitinib have shown that toxicity driven dosing is a feasible and effective approach [48, 49]. However, the therapeutic index of cabozantinib appears to be narrower compared to other VEGFR-inhibitors and tolerability and toxicity may partly overlap, which makes toxicity driven dosing a less feasible approach. In addition, the long half-life of cabozantinib may lead to slow recovery from toxicity. Still, there appears to be a sweet spot for cabozantinib in which efficacy can be balanced with manageable toxicity, preferable by combining both cabozantinib exposure and clinical evaluation to avoid excessive toxicity.

In a prior exposure–response analysis performed by Lacy and colleagues, increased efficacy was observed for patients with an cabozantinib exposure above 750 ng/mL [24]. Similarly, the CABOREAL study also reported a slightly longer OS for patients who initiated treatment with the 60 mg dose compared to patients with a lower starting dose (15.4 months versus 11.8 months, respectively) [35]. Unexpectedly, we did not observe an association between a 60 mg starting dose or a high start exposure and improved PFS in our study. In fact, we observed a numerically but not significantly longer PFS in patients with an average exposure below the median exposure. This observation may indicate that tolerability is at least as important for long term treatment benefit as sufficient exposure. This unexpected result may partly have been caused by treatment adherence in patients suffering from adverse events. Unfortunately, we were not able to collect data on treatment adherence, but as a higher cabozantinib exposure results in increased toxicity this might also compromise treatment adherence and thereby negatively affect treatment efficacy.

The majority of patients in our study had an average cabozantinib exposure below the previously proposed target exposure of ≥ 750 ng/mL, while this target exposure is already adjusted compared to the original C_min_ of 1125 ng/ml in the registration trial. Fifty-four percent of the patients in our cohort started with a dose of ≤ 40 mg instead of the recommended 60 mg dose by the label, which can partly explain this lower exposure. Still, 58% of patients needed a dose reduction relative to their starting dose. Patients who needed a dose reduction had a significantly higher cabozantinib exposure compared to the patients without a dose reduction (831 ng/mL vs. 569 ng/mL, *p* = 0.001). In addition, the exposure at the final dose level was significantly lower in patients who required a dose reduction compared to those who did not require a dose reduction (522 ng/mL vs. 740 ng/mL, *p* = 0.001). This may suggest a higher cabozantinib sensitivity in patients who needed a dose reduction, which may also explain the observed increase in PFS in these patients. Future studies should therefore include details on the toxicities necessitating dose reductions in order to evaluate them as potential pharmacodynamic markers of response. However, the difference in exposure could also be the result of the available dosage strengths and recommended dose adjustments according to the drug label, i.e. the dose reduction from 40 to 20 mg being too substantial. More gradual dose alterations based on patients individual cabozantinib exposure and the use of alternative dosing schedules might help individual patients to achieve their optimal individual exposure and increase treatment benefit.

Based on the observations in the current study, we developed an algorithm for treatment optimisation, incorporating both cabozantinib exposure and clinical parameters. As the 40 mg dose has a higher chance compared to the 60 mg dose to result in an exposure in the tolerable window of 500–800 ng/mL, this might be considered as the preferable starting dose. From this starting point, the dose can be gradually adjusted with alternative dosing schedules based on both the measured exposure and clinical symptoms and parameters. Our strategy enables to reach sufficiently high cabozantinib exposure while avoiding excessive toxicity and thereby potentially adherence issues. Although we did not observe a difference in PFS between patients who had an exposure > 750 ng/mL compared to those with a lower exposure in our exploratory analysis, evaluation of this approach in a larger and less heterogenous cohort is warranted to confirm comparable or improved efficacy. In addition, the combined approach in our algorithm may have some advantages over the toxicity driven dosing approach. For patients whose disease progresses during cabozantinib treatment, a higher cabozantinib exposure may be required for treatment response. For axitinib, sunitinib and pazopanib, dose escalation has been shown to be an effective strategy, leading to a decrease in tumour burden [50, 51]. For cabozantinib, a similar approach may apply and our algorithm may help select patients in whom dose escalation can be performed safely with respect to the narrow therapeutic window of cabozantinib. Also, for some VEGFR-Inhibitors, a clear relationship between certain toxicities and drug exposure and/or treatment response is lacking [23, 52, 53]. By using the combined approach, a more informed decision can be made between dose reductions or switch of therapy, avoiding futile dose reductions and hence delay of effective treatment in patients with low exposure.

Limitations of our study include the retrospective nature of the analysis and potential selection bias. As we only included patients with a measured cabozantinib exposure at steady-state, this could have excluded patients who had progressed or had severe toxicity before their cabozantinib levels were measured. Moreover, as we measured the cabozantinib exposure in patients who were already receiving cabozantinib as well as starting patients when we implemented the cabozantinib assay, patients with treatment benefit may have been overrepresented in our selection. In addition, evaluation and radiology review was not standardized and patients may have been treated beyond progression. Nevertheless, the observed PFS was comparable to a previously reported real-world analysis [42]. Another important limitation to mention is that the observed increased survival in patients with a dose reduction can also be the result of guaranteed time bias, i.e. the chance of a dose reduction increases with increased treatment duration. However, the median time to first dose adjustment was within 8 weeks and therefore the long term effect of cabozantinib exposure on PFS warrants further research. Furthermore, the number of patients in our analysis was relatively small and consisted of multiple histological subtypes and IMDC risk groups. Patients with more indolent disease may have had a higher a priori chance of being treated with cabozantinib in later treatment lines and may have been overrepresented in our analysis. The presence of a more sensible phenotype may thereby have diluted the cabozantinib exposure–response relationship. However, this complex heterogeneity is unavoidable in real world studies and yet representative for patients with mRCC in clinical practice.

Nevertheless, the current study is one of the first studies that described cabozantinib exposure in relation to efficacy and toxicity in routine care. Future studies should focus on further elucidating the exposure–response relationship, implementing dose and schedule optimisation and identifying (bio)markers that help upfront selection of patients who will benefit from cabozantinib treatment. This approach aligns with the new paradigm for targeted therapies, which seeks the dose required rather than the maximum tolerated dose [54].

## Conclusion

In this study, we did not observe a clear relationship between cabozantinib exposure and PFS. However, an increased PFS was observed in patients who required a dose reduction, which may indicate the need for further deciphering the exposure response relationship. Based on our explorative analyses, a starting dose of 40 mg will most likely result in a tolerable exposure. Subsequently, cabozantinib treatment can be further optimised based on a combination of clinical parameters and the measured cabozantinib exposure.

## Supplementary Information

**Additional file 1.**

## Data Availability

The datasets generated and analysed for the current study are available from the corresponding author on reasonable request.
