# Supplementary material for: Exposure–response analyses of cabozantinib in patients with metastatic renal cell cancer
Source: BMC Cancer. 2022 Mar 2;22:228. doi: 10.1186/s12885-022-09338-1 (PMC8892746; doi:10.1186/s12885-022-09338-1)
Supplement: Supplementary file 1 — Additional file 1. [file 12885_2022_9338_MOESM1_ESM.docx]

Supplementary Files

Index

[**Supplementary Method:** cabozantinib dose extrapolation 2](#_Toc86586805)

[**Supplementary Figure 1A:** Kaplan-Meier curve of PFS for patients with an average exposure above and below the proposed target of >750 ng/mL 3](#_Toc86586806)

[**Supplementary Figure 1B**: Kaplan-Meier curve of PFS for patients with a start exposure above and below >750 ng/mL 4](#_Toc86586807)

[**Supplementary Figure 1C**: Kaplan-Meier curve of PFS for patients with a start dose of 60 mg and 40 mg 5](#_Toc86586808)

[**Figure 1D:** Kaplan-Meier curve of progression free survival for patients with an average exposure above and below 750 ng/mL during the first 90 days of treatment 6](#_Toc86586809)

[**Supplementary Table 1:**Univariable and multivariable Cox regression analysis for PFS 7](#_Toc86586810)

[**Supplementary Figure 2A**: Kaplan-Meier curve of overall survival (OS) for patients according to the IMDC risk groups 8](#_Toc86586811)

[**Supplementary Figure 2B:** Kaplan-Meier curve of OS for intermediate IMDC risk group patients with an exposure above and below the median average exposure of 572 ng/mL 9](#_Toc86586812)

[**Supplementary Figure 2C:** Kaplan-Meier curve of overall survival for poor IMDC risk group patients with an exposure above and below the median average exposure of 572 ng/mL 10](#_Toc86586813)

[**Supplementary Figure 2D:** Kaplan-Meier curve of OS for intermediate IMDC risk group patients with and without a dose reduction relative to the start dose 11](#_Toc86586814)

[**Supplementary Figure 2E:** Kaplan-Meier curve of OS for poor IMDC risk group patients with and without a dose reduction relative to the start dose 12](#_Toc86586815)

**Supplementary Method:** cabozantinib dose extrapolation

Cabozantinib exposures at start and at best tolerated dose level, respectively, were determined using extrapolation of the dose-normalized average exposure based on all available cabozantinib C_min_ measurements per patient. Cabozantinib shows dose-proportional exposure over the range of 20 to 140 mg, which supports the use of this approach. For each patient, the average dose-normalized cabozantinib C_min_ exposure per mg cabozantinib was calculated as follows: First, each available cabozantinib C_min_ measurement at steady-state was dose-normalised by dividing it by the administered dose of cabozantinib used at the moment of measurement (cabozantinib exposure/administered dose of cabozantinib in mg = ng/mL per mg of cabozantinib). Subsequently, the average dose-normalised exposures was calculated per patient (sum of dose-normalised exposure levels / number of measurements). This average concentration was thereafter multiplied by the specific dosages of interest e.g. starting dose, best tolerated dose level. The average cabozantinib exposure over the course of treatment was estimated based on steady-state exposures. This estimate was calculated by multiplying the number of days at each dose level of cabozantinib with the corresponding milligrams of cabozantinib. The sum of the amount of cabozantinib was subsequently divided by the duration of treatment in days to yield the average amount of cabozantinib in mg per day. The average cabozantinib exposure was thereafter calculated by multiplying this average amount of cabozantinib with the average exposure/mg cabozantinib.

## **Supplementary Figure 1A:** Kaplan-Meier curve of PFS for patients with an average exposure above and below the proposed target of >750 ng/mL


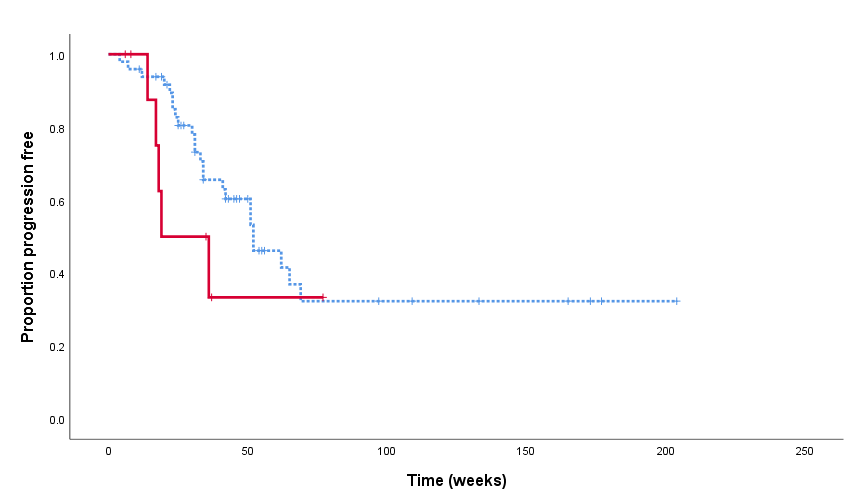


Median PFS was 19 weeks (95% CI:0-40) in the group with an average exposure equal or above 750 ng/mL (solid red line) vs 52 weeks (95% CI: 34-70) in the group with an average exposure below 750 ng/mL (dotted blue line) (*P*=.2)

## **Supplementary Figure 1B**: Kaplan-Meier curve of PFS for patients with a start exposure above and below >750 ng/mL


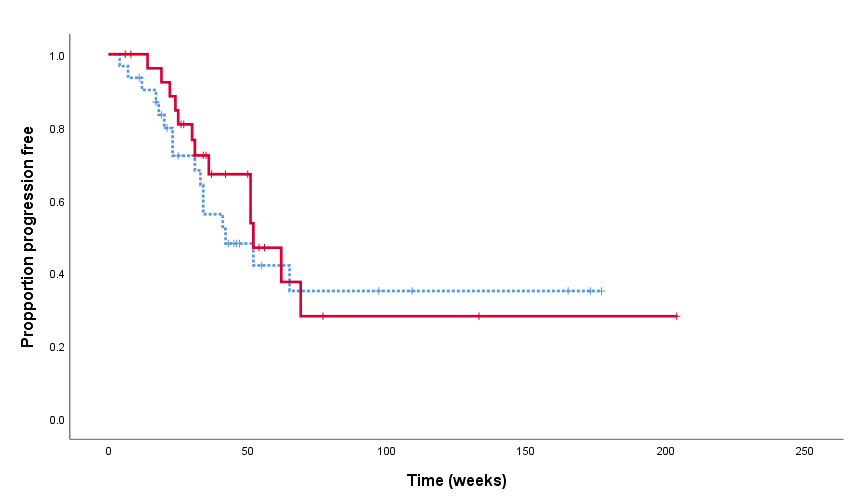


Median PFS was 52 weeks (95% CI:32-72) in the group with a start exposure equal or above 750 ng/mL (solid red line) vs 42 weeks (95% CI:17-66) in the group with a start exposure below 750 ng/mL (dotted blue line) (*P*=.6)

## **Supplementary Figure 1C**: Kaplan-Meier curve of PFS for patients with a start dose of 60 mg and 40 mg


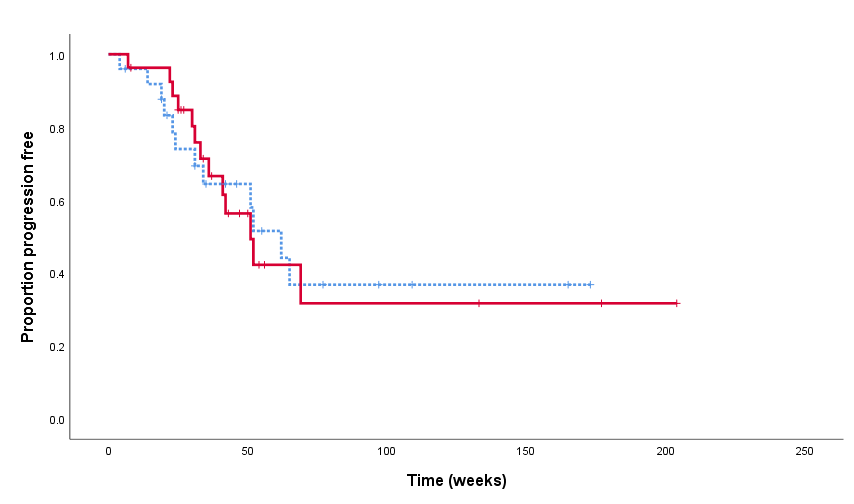


Median PFS was 51 weeks (95% CI:35-67) in the group with a starting dose of 60 mg (solid red line) vs 62 weeks (95% CI:43-81) in the group with a starting dose of 40 mg (dotted blue line) (*P*=.9)

## **Figure 1D:** Kaplan-Meier curve of progression free survival for patients with an average exposure above and below 750 ng/mL during the first 90 days of treatment

**
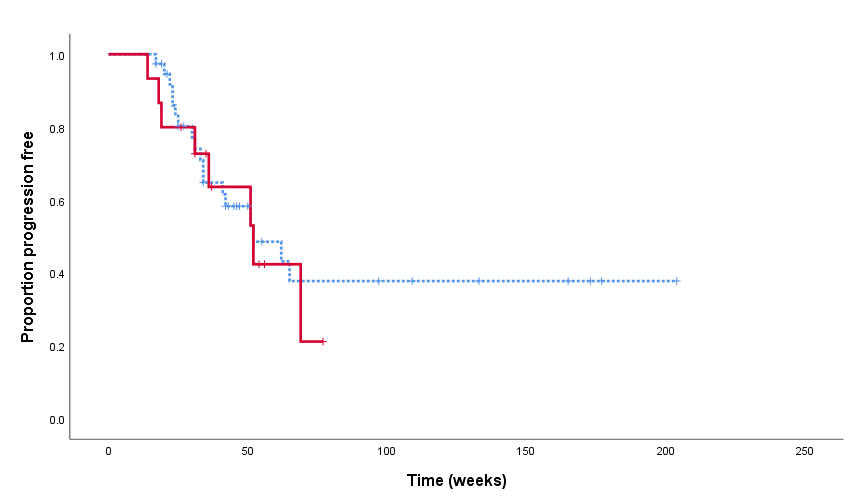
**

For this analysis only patients with an cabozantinib treatment duration > 90 days were included (n=53). Average cabozantinib exposure was calculated over the first 90 days of treatment. Median PFS was 52 weeks (95% CI: 30-74) in the group with an average exposure ≥750 ng/mL over the first 90 days (solid red line, n=15) vs 52 weeks (95% CI:27-77) in the group with an average exposure below 750 ng/mL(dotted blue line, n=38) (*P*=.7)

## **Supplementary Table 1:**Univariable and multivariable Cox regression analysis for PFS

##

|  |  | n | Univariate | | | Multivariate | | |
| --- | --- | --- | --- | --- | --- | --- | --- | --- |
| Variable | Subgroup |  | HR | 95%CI | P value | HR | 95%CI | P value |
| IMDC | favourable | 6 | - | - | - | - | - | - |
|  | intermediate | 36 | 2.95 | 0.39-22.35 | .295 | 2.93 | 0.47-28.30 | .219 |
|  | poor | 17 | 5.61 | 0.73-43.25 | .098 | 4.98 | 0.63-39.21 | .127 |
| Prior lines of treatment | < 2 | 22 | - | - | - | - | - | - |
|  | ≥ 2 | 37 | 0.49 | 0.23-1.03 | .061 | 0.59 | 0.27-1.28 | .183 |
| C_min_ over duration of treatment | < 572 ng/mL | 29 | - | - | - | - | - | - |
|  | ≥ 572 ng/mL | 30 | 2.04 | 0.96-4.31 | .062 |  |  |  |
| Dose reduction* | No | 25 | - | - | - | - | - | - |
|  | Yes | 34 | 0.47 | 0.15-0.67 | .002 | 0.32 | 0.14-0.70 | .004 |

* dose reduction relative to the starting dose

Abbreviations; IMDC, International Metastatic RCC Database Consortium; C_min,_, cabozantinib trough concentrations levels

## **Supplementary Figure 2A**: Kaplan-Meier curve of overall survival (OS) for patients according to the IMDC risk groups


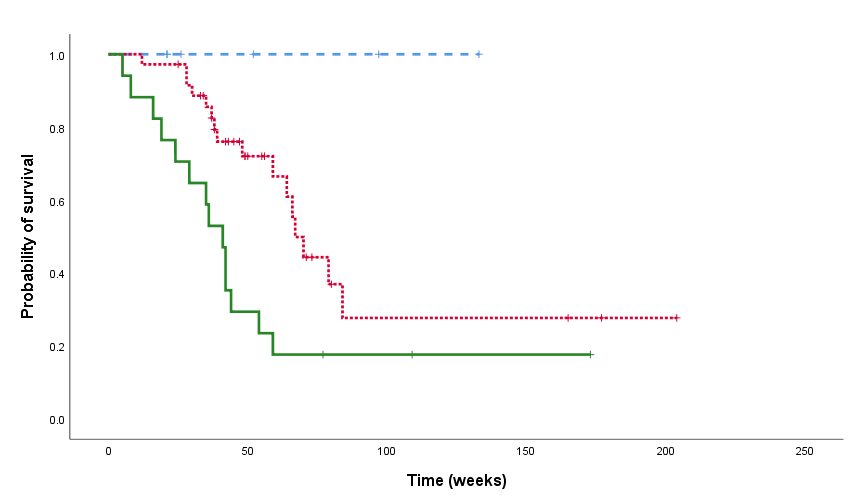


Median OS was not reached for favourable IMDC risk patients (dashed blue line), 67 weeks (95%CI:59-75) in intermediate IMDC risk patients (dotted red line) and 41 weeks (95%CI: 34-48) in poor IMDC risk patients (solid green line).

## **Supplementary Figure 2B:** Kaplan-Meier curve of OS for intermediate IMDC risk group patients with an exposure above and below the median average exposure of 572 ng/mL


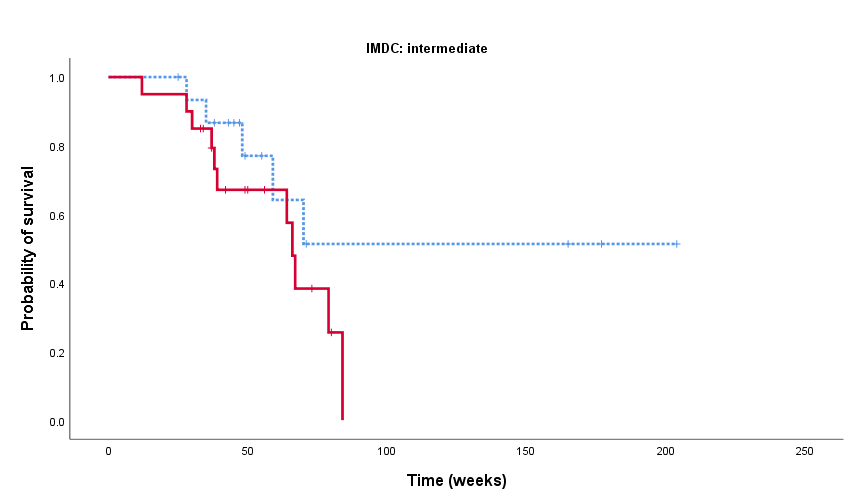


Median OS was 66 weeks (95% CI:62-70) in the group with an average exposure equal or above the median of 572 ng/mL (solid red line) vs not reached in the group with a start exposure below the median average exposure of 572 ng/mL (dotted blue line) (*P*=.15)

**Supplementary Figure 2C:** Kaplan-Meier curve of overall survival for poor IMDC risk group patients with an exposure above and below the median average exposure of 572 ng/mL

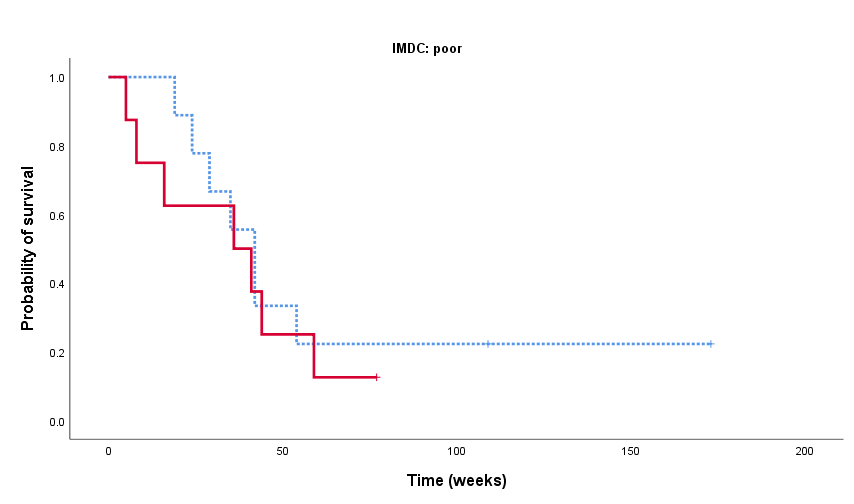


Median OS was 36 weeks (95% CI:1-71) in the group with an average exposure equal or above the median of 572 ng/mL (solid red line) vs 42 weeks (95%CI: 32-52) in the group with a start exposure below the median average exposure of 572 ng/mL (dotted blue line) (*P*=.6)

## **Supplementary Figure 2D:** Kaplan-Meier curve of OS for intermediate IMDC risk group patients with and without a dose reduction relative to the start dose


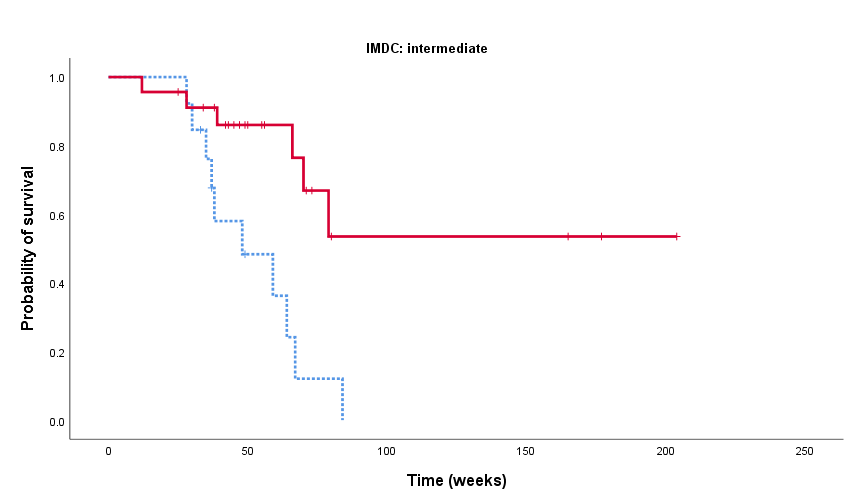


Median OS was not reached in the group with a dose reduction (solid red line) vs 48 weeks (95% CI: 20-76) weeks in the group without dose reduction (dotted blue line) (*P*=.002)

## **Supplementary Figure 2E:** Kaplan-Meier curve of OS for poor IMDC risk group patients with and without a dose reduction relative to the start dose


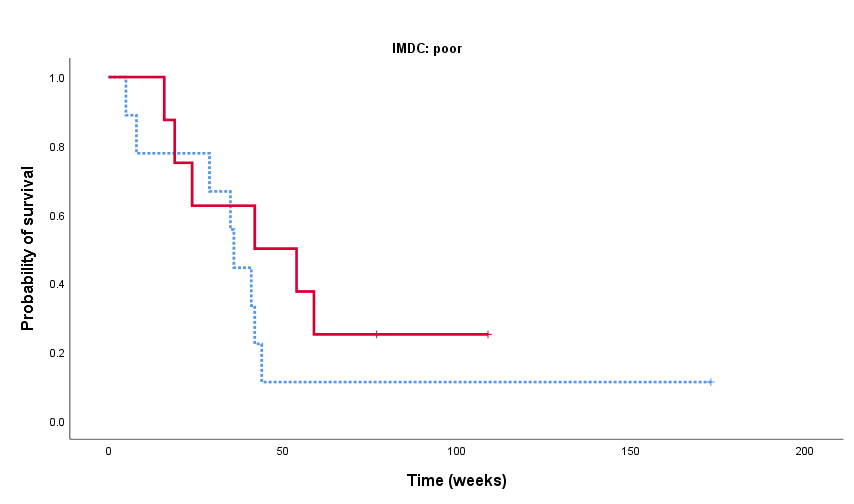


Median OS was 42 (95% CI: 0-84) weeks in the group with a dose reduction (solid red line) vs 36 (95% CI: 33-39) weeks in the group without dose reduction (dotted blue line) (*P*=.30)
